# Lipopolysaccharide Induces Up-Regulation of TGF-α through HDAC2 in a Rat Model of Bronchopulmonary Dysplasia

**DOI:** 10.1371/journal.pone.0091083

**Published:** 2014-03-04

**Authors:** Wensi Ni, Ning Lin, Hua He, Jianxing Zhu, Yongjun Zhang

**Affiliations:** 1 XinHua Hospital, Shanghai Jiaotong University School of Medicine, Shanghai, China; 2 MOE and Shanghai Key Laboratory of Children's Environmental Health, Shanghai, China; Southern Illinois University School of Medicine, United States of America

## Abstract

Bronchopulmonary dysplasia (BPD) is characterized by alveolar simplification with decreased alveolar number and increased airspace. Previous studies suggested that transforming growth factor-α (TGF-α) may contribute to arrested alveolar development in BPD. Histone deacetylases (HDACs) control cellular signaling and gene expression. HDAC2 is crucial for suppression of inflammatory gene expression. Here we investigated whether HDAC2 was involved in the arrest of alveolarization, as well as the ability of HDAC2 to regulate TGF-α expression in a rat model of BPD induced by intra-amniotic injection of lipopolysaccharide (LPS). Results showed that LPS exposure led to a suppression of both HDAC1 and HDAC2 expression and activity, induced TGF-α expression, and disrupted alveolar morphology. Mechanistic studies showed that overexpression of HDAC2, but not HDAC1, suppressed LPS-induced TGF-α expression. Moreover, the HDAC inhibitor TSA or downregulation of HDAC2 by siRNA both significantly increased TGF-α expression in cultured myofibroblasts. Finally, preservation of HDAC activity by theophylline treatment improved alveolar development and attenuated TGF-α release. Together, these findings indicate that attenuation of TGF-α-mediated effects in the lung by enhancing HDAC2 may have a therapeutic effect on treating BPD.

## Introduction

Bronchopulmonary dysplasia (BPD) is characterized by arrested alveolar developmental with decreased saccular airway branching and fewer, larger alveoli, leading to reduced surface-to-volume ratio and respiratory insufficiency [1], [2]. Studies have shown that inflammation increases the risk of BPD in the infant before birth, as suggested by the positive correlation between chorioamnionitis and adverse lung development [3]–[5]. Better understanding of the mechanisms by which inflammation disrupts lung development may provide insight into the pathogenesis of BPD and offer avenues for therapeutic development.

The definitive alveoli are established during development of the outgrowth of secondary septa from the primary septa present in newborns. The growth of secondary septa leads to saccule subdivision and enlarges the gas-exchanging surface [6], 7. Elastin is required for initiation and progression of alveolization, which is synthesized and secreted by alveolar myofibroblasts [8]. It is suggested that alveolar myofibroblasts may play an important role in alveolar maturation. PDGF-A-null mice had a complete loss of myofibroblasts and exhibited defects in alveolization at birth [9]. Transforming growth factor-α (TGF-α) is a member of the epidermal growth factor family that binds to and activates EGF receptor (EGFR). The TGF-α/EGFR signaling pathway plays a central role in lung development [10]. TGF-α has been suggested as the key stimulus for the stabilizing myofibroblasts polarity, which is critical to secondary septation and may contribute to arrested alveolar development in BPD [11]. More specifically, the expression of TGF-α/EGFR increased in the lungs of infants with BPD [12]. Additionally, overactivation of EGFR in TGF-α transgenic mice led to pathological changes similar to those in the lungs of BPD patients [13]. Our previous studies demonstrated that lipopolysaccharide (LPS) increased TGF-α expression in myofibroblasts [11]. However, a further regulatory mechanism at the transcriptional level requires clarification.

Histone deacetylases (HDACs) determine the acetylation status of histones and thereby controls the regulation of gene expression. HDACs form a large family, of which class I HDACs, including the closely related proteins HDAC1 and HDAC2, show the strongest histone deacetylase activity. HDAC2 is crucial for embryonic development and affects cytokine signaling relevant for immune responses [14]. HDAC2 suppresses inflammatory gene expression and appears to be a key factor in the development of inflammatory airway disease [15]. Theophylline is a bronchodilator, which is also described as an effective agonist of HDAC. Several studies have shown that low-dose theophylline exerts an anti-inflammatory effect through increasing activation of HDAC [16], [17]. In addition, LPS decreased the mRNA expression of HDAC2 in lung fibroblasts [18]. Reduction of HDAC2 activity in the lung is correlated with increased expression of IL-8 in chronic obstructive pulmonary disease (COPD) [19], [20], but its potential role during the pathogenesis of BPD remains unknown.

In this paper, we attempt to address whether HDAC2 is involved in the LPS-induced arrest of alveolarization and the effect of HDAC2 on the expression of TGF-α. We found that LPS exposure led to a suppression of both HDAC1 and HDAC2 expression and activity, induced TGF-α expression, and disrupted alveolar morphology. Overexpression of HDAC2, but not HDAC1, suppressed LPS-induced TGF-α expression. Moreover, the HDAC inhibitor TSA or down-regulation of HDAC2 by siRNA both significantly increased TGF-α expression. Finally, preservation of HDAC activity by theophylline treatment improved alveolar development and attenuated TGF-α release. This study provides the first insight into the roles and potential mechanisms of HDAC2 in the pathogenesis of BPD.

## Materials and Methods

### Ethics statement

All animal experiments were approved by the ethics committee of the XinHua Hospital, Shanghai Jiaotong University School of Medicine. All experimental procedures were performed in accordance with the “Guide for Care and Use of Laboratory Animals” published by the National Institutes of Health. Sprague Dawley rats were anesthetized by intraperitoneally injection of 10% chloral hydrate at a dose of 3.5 ml/kg body weight. All efforts were made to minimize suffering.

### Animal studies

Pregnant rats (purchased from the Shanghai Laboratory Animal Center) were randomized into three groups (n = 3 in each group): (1) no treatment group; (2) intra-amniotic LPS injection group (200 µg/ml, 5 µl); (3) intra-amniotic saline injection group (5 µl). For the establishment of chorioamnionitis, pregnant rats on embryonal day 16.5 (E16.5) were injected with purified LPS from *E. coli* via phenol-extraction and ion-exchange purification. Pups in the LPS group were divided into two groups immediately after birth: one group was injected with theophylline (20 mg/kg/day) and the other group was injected with vehicle. Theophylline was dissolved in saline to at a final concentration of 5 mg/ml, and injected subcutaneously into the neck of the pups.

### Tissue preparation

A thoracotomy was conducted on neonatal rats on postnatal day 3 (P3), P7 and P14 separately (n = 3–5 in each group). The right primary bronchi were ligated and inflated with 4% paraformaldehyde for morphometric analysis. The right lungs were removed, frozen in liquid nitrogen and stored at −80°C for further experiments.

### Morphometric analysis

Tissue was immersion-fixed overnight, dehydrated through graded alcohols, washed in 100% ethanol and CitriSolv, and embedded in paraffin. Serial 5 μm-thick sections were stained with hematoxylin and eosin (H&E). At each time point 5 random non-overlapping fields per pup and 3 pups per experimental group were analyzed for morphometric examinations. Terminal air spaces and alveolar secondary crests were counted manually in each field.

### Myofibroblast culture

Myofibroblast culture was prepared in accordance with previous studies [11]. Human embryonic lung fibroblasts (HFL-I) were purchased from Cell Bank, Chinese Academy of Sciences. HFL-I cells were cultivated in α-MEM medium (invitrogen) with 10% FBS (invitrogen), 100 units penicillin/ml, and 100 μg streptomycin/ml at 37°C in humidified 5% CO_2_. After removing the medium, cells were incubated in α-MEM medium supplemented with 1% FBS, in the presence of BSA alone (control) or LPS (1 μg/ml) for 1 h or 16 h. In some experiments, cells were pretreated with theo (theophylline, 10 μM) or TSA (Trichostatin A, 10 ng/ml) for 30 min before LPS-treatment. All of the reagents mentioned were purchased from Sigma (USA).

### Plasmid transfection

The pPGK-HDAC2 RNAi vector and GFP-tagged wild-type HDAC1 were provided by Dr J Song (Institute of Biochemistry and Cell Biology, Chinese Academy of Sciences, Shanghai, China) [21]. The pcDNA-HDAC2 expression vector was kindly provided by Dr E. Seto (Moffitt Cancer Center, University of South Florida, USA). HFL-I cells expressing vector were grown in Dulbecco's modified Eagle's medium supplemented with 10% bovine calf serum. Transfections were performed using lipo2000 (Invitrogen) according to the manufacturer's recommendations. Cells were transfected with the pcDNA-HDAC2 and GFP-tagged wild-type HDAC1 for 24 h before exposure to LPS for 1 or 16 hours. Cells were transfected with pPGK-HDAC2 RNAi vector and kept in culture for 48 hours.

### Nuclear-cyotosolic fractionation

Myofibroblasts were harvested and re-suspended in 5 pellet volumes of cold buffer A (Hepes-KOH 10 mM, KCL 10 mM, MgCL_2_ 5 mM, EDTA 0.5 mM, EGTA 0.5 mM), and put on ice for 15 min. Cells were flushed through a syringe until the plasma membrane was lysed as indicated by trypan blue staining, and spun down at low speed (1000 g) for 5 min at 4°C. Then the supernatant (cytosol) was transferred to new EP tubes and spun at high speed for 10 min to clear debris. Next, the pellet was washed (nuclear) in 2 pellet volumes of cold buffer B (Hepes-KOH 10 mM, NaCL 0.42 M, glycerol 2.5% v/v, MgCL_2_ 1.5 mM, EDTA 0.5 mM, EGTA, 0.5mM, DTT 1 mM), and nuclear fractions were rotated at 60 rpm at 4°C for 30 min. Finally, a high speed spin was used to clear nuclear debris. The Bradford assay (Pierce, USA) was used to quantitate the protein concentration in each fraction.

### HDAC1 and HDAC2 activity measurement

HDAC1/2 activity was measured using a colorimetric assay kit (Genmed, USA) according to the manufacturer's manual as described [22]. Briefly, nuclear extract samples were incubated with HDAC1/2 colorimetric substrate (Boc-Lys (Ac)-pNA) with specific inhibitors (MS275 or apicidin). The deacetylation sensitizes the substrate so subsequent treatment with the aminopeptidase produces a fluorophore that can then be measured using a fluorescence reader.

### Western blotting Analysis for Determining HDAC1/2

Nuclear proteins were extracted from lung tissue of neonatal rats. Myofibroblasts were treated as described above. For HDAC1/2 assays, 20 μg of isolated soluble proteins were electrophoresed on 10% PAGE gels and transblotted onto nitrocellulose membranes. HDAC1/2 protein was detected with goat anti-human anti-HDAC1/2 antibody (Santa Cruz). Densitometric analysis was performed with Bio-Rad ChemiDoc XRS+ software, and the relative ratio to actin (Santa Cruz) expression was calculated in each sample.

### RNA isolation and real time PCR for TGF-α

Total RNA was isolated with Trizol (Invitrogen, USA) and dissolved in RNase-Free water. The resulting DNA-free RNA was measured by UV spectroscopy at 260 nm. Total RNA from each sample was reverse transcribed to cDNA via the PrimeScriptTM RT reagent Kit (Takara). The SYBR Premix Ex TaqTM kit (Takara) was used for quantitative real-time RT-PCR analysis. The primers were designed using Primer Express software (Applied Biosystems) and synthesized by Takara. The following gene specific primers were used: TGF-α (forward: 5′-ctggctgtcctcattatcacct-3′; reverse: 5′-aaattcctcctctgggatcttc-3′); GAPDH (forward: 5′-gcaagttcaacggcacag-3′; reverse: 5′-gccagtagactccacgacat -3′). Real time PCR was performed on the ABI Prism 7500 sequence detection PCR machine (Applied Biosystems, USA) according to manufacturer's protocol. Relative differences in gene expression between groups were calculated using the Ct method.

### Enzyme-linked immunosorbent assay (ELISA)

The concentration of TGF-α in cell culture supernatants was determined by ELISA (R&D Systems, USA) as described in the manufacturer's instructions.

### Statistical analyses

The results are expressed as the mean ± SE of three or more independent experiments. Statistical analysis was performed using the software SPSS 16.0. Student's t-test and one-way ANOVA were used to analyze the statistical significance. A value of *P*<0.05 was considered statistically significant.

## Results

### Intra-amniotic LPS injection disrupted alveolar development

**Figure 1:**
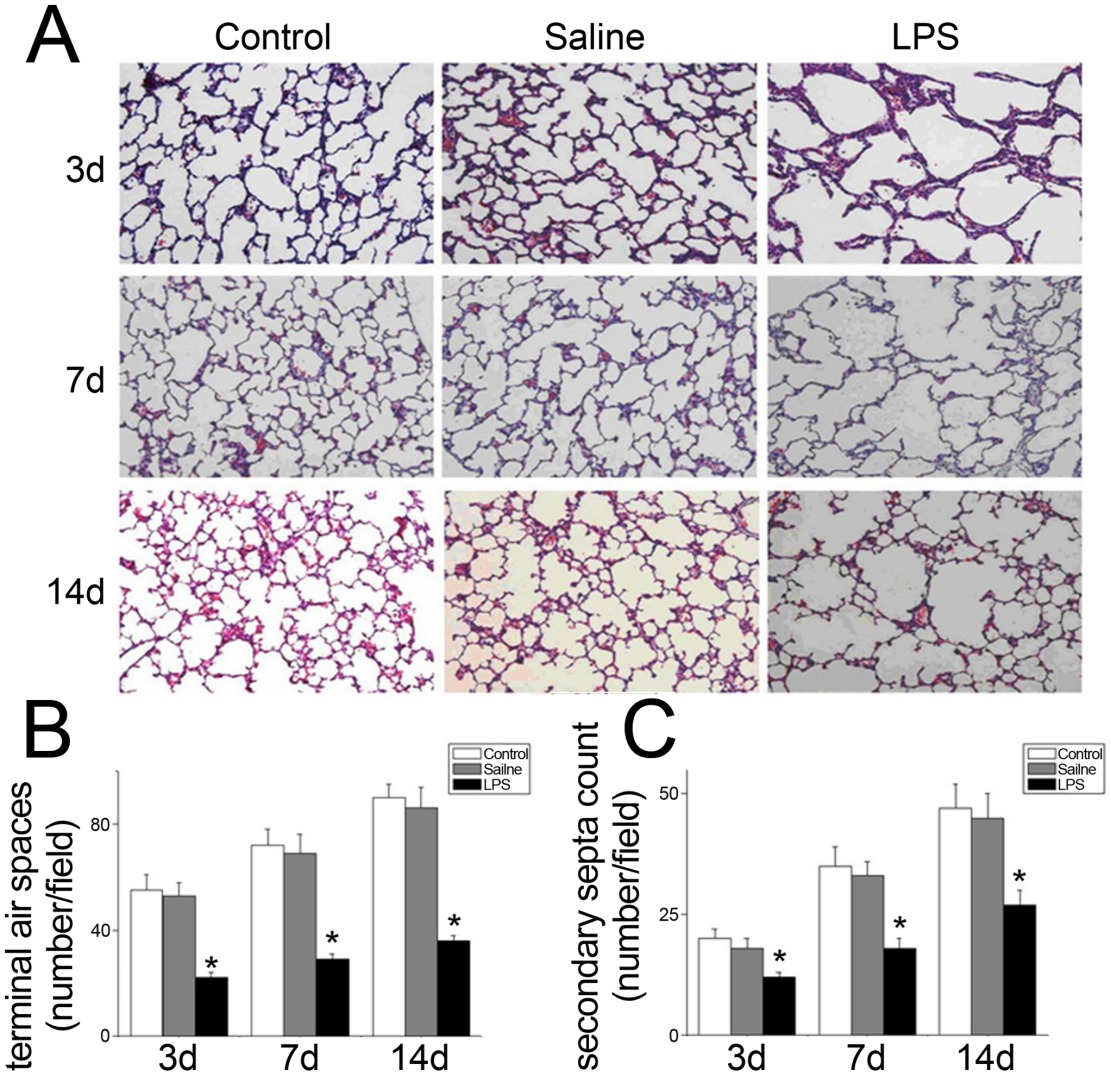
LPS injection disrupted alveolar development in neonatal rats. E16.5 rats were injected with LPS or saline via the amniotic sac. Rats with no administration were used as control. The neonatal rats were thoracotomized and the right lungs were removed at 3, 7 and 14: Lung tissues were stained with H&E. B: Morphometric measurements showed that terminal air spaces were significantly fewer in the LPS-treated rats at all the three time points (3, 7 and 14 days). C: The secondary septa count was significantly less after exposure to LPS for 3, 7 and 14 days. Results from 3 separate experiments were pooled and the mean ± SE was calculated. * *P*<0.05, significantly different from saline or control groups.

Pregnant rats were injected with LPS or saline in the amniotic sac at E16.5. Rats with non-injection were used as control. No fetal death occurred in the control group and almost all rat pups survived until the end of the experiment. Pups treated with intra-amniotic LPS injection showed a survival rate of approximately 80%. Most fatalities happened before birth. Lung tissue samples from the LPS-injected group displayed simplified alveolar structure, as indicated by the enlarged alveoli with decreased terminal air spaces and secondary septa (Fig. 1A). Morphometric measurements revealed that the terminal air space count was significantly decreased in the LPS-treated group compared with the saline-treated group or the control group at all time points investigated (3 days; 7 days; and 14 days ; Fig. 1B). LPS treatment significantly decreased secondary septa at all three time points (Fig. 1C).

### LPS exposure elevated the expression of TGF-α both *in vivo* and *in vitro*

**Figure 2:**
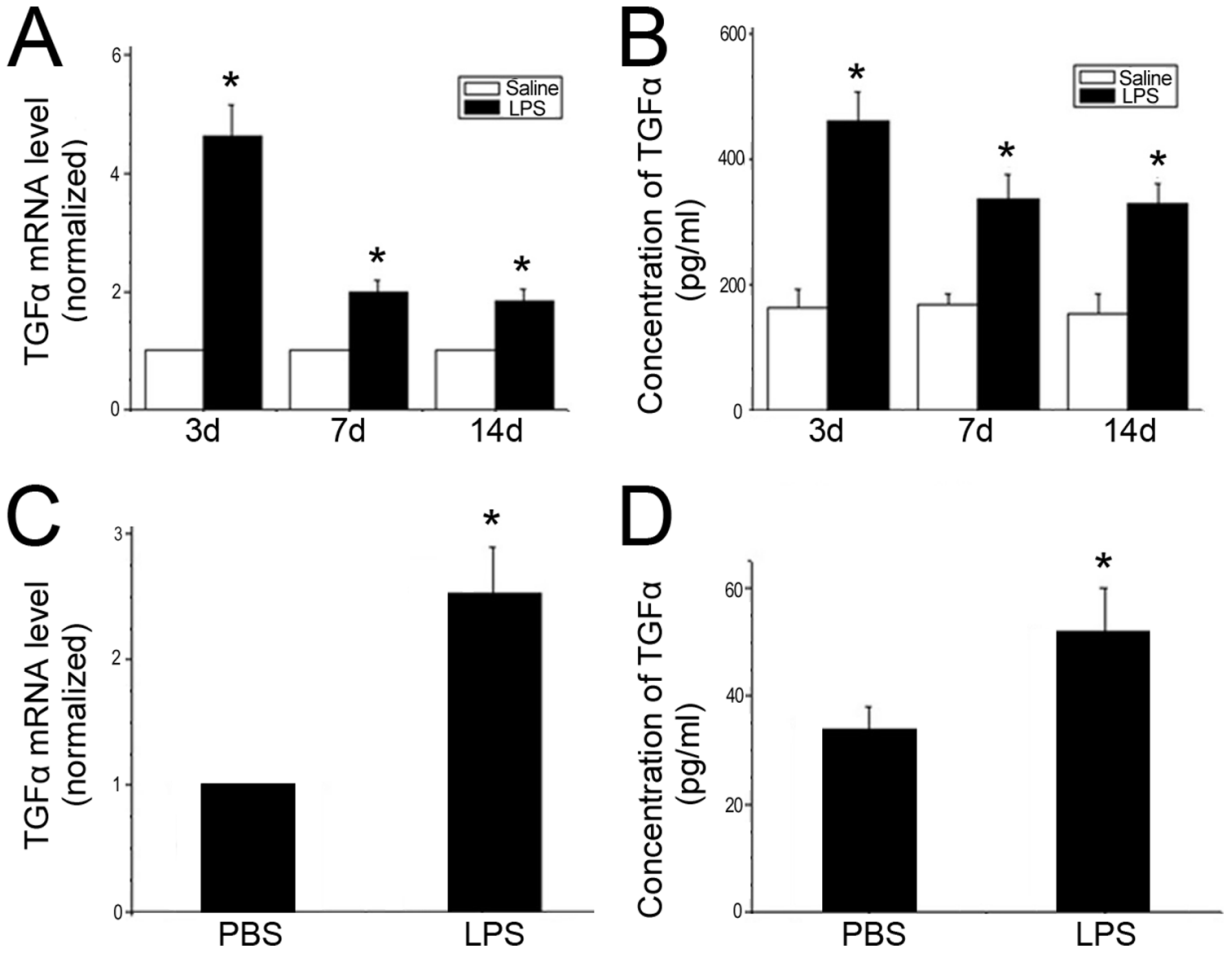
LPS elevated the expression of TGF-α both in *vivo* and *vitro*. A: Pregnant rats were injected with LPS or saline as control. TGF-α mRNA expression was upregulated in lungs of antenatal LPS-treated rats on day P3, P7 and P14. B: TGF-α protein was significantly elevated in lungs of antenatal LPS-treated rats. C: Myofibroblasts were treated with LPS (1 μg/ml) or PBS for 1 h. TGF-α mRNA expression was upregulated by LPS in cell culture. D: TGF-α level in cell culture supernatants (1 μg/ml LPS for 16 h) was elevated compared to that in PBS group. Values are means ± SE. Each experiment was repeated at least three times. * *P*<0.05, significantly different from the saline or PBS group.

TGF-α mRNA levels in rat lungs were increased in the LPS injection group by 4.6-, 2.2- and 1.8-fold at day 3, 7 and 14, respectively (Fig. 2A). Similar results were found in TGF-α protein levels detected by ELISA. Protein levels were elevated 2.87-, 2.18- and 2.11- fold on day 3, 7 and 14 compared to the saline-treated group (Fig. 2B). TGF-α mRNA level was increased by 2.51- folds (Fig. 2C) and the TGF-α protein level increased by 52.9% in myofibroblasts treated with LPS (Fig. 2D). These results suggest that LPS could induce the expression of TGF-α.

### LPS exposure suppressed the expression and activity of HDAC1 and HDAC2

**Figure 3:**
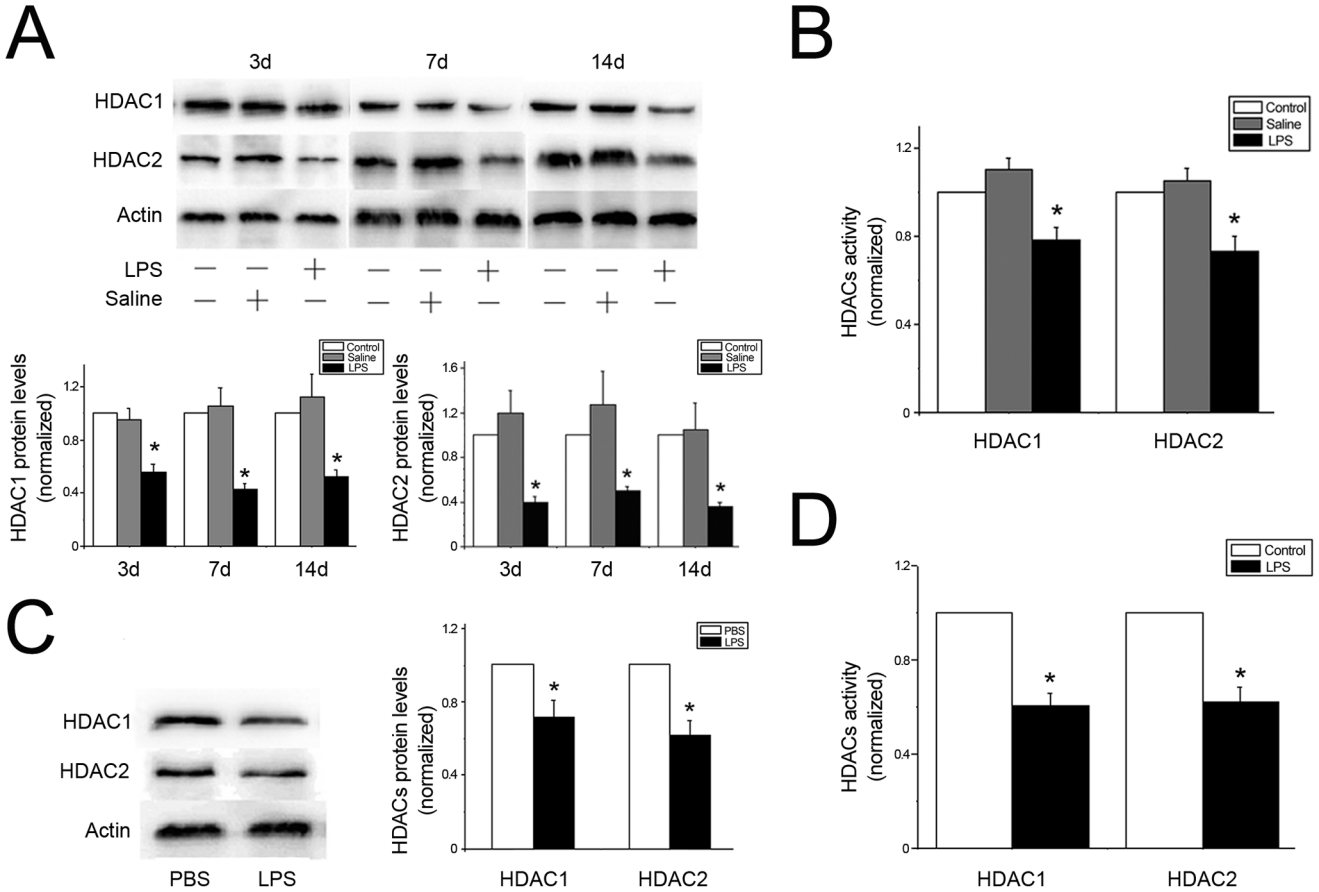
The expressions and activity of HDAC1/2 were downregulated by LPS exposure. A: HDAC1/2 protein expressions were significantly reduced in rats exposed to LPS for 3, 7, and 14 days. B: The activities of HDAC1/2 were also significantly decreased in rats treated with LPS for 7 days. C: HDAC1/2 protein expressions were significantly reduced in myofibroblasts exposed to LPS for 16 h. D: The activities of HDAC1/2 were also significantly decreased in myofibroblasts exposed to LPS for 1h. Results from 3 separate experiments were pooled and the mean ± SE was calculated. * *P*<0.05, significantly different from control group.

In the lung, HDAC1/2 deficiency leads to a block in airway epithelial cell development [23]. Here the results showed that the HDAC1/2 protein level was significantly decreased in LPS-injected rats at day 3, 7 and 14. The protein levels of HDAC2 decreased by 60%, 50% and 64% at day 3, 7 and 14, respectively, compared with the control group (Fig. 3A). The protein level of HDAC1 showed a similar trend (Fig. 3A). In rat lung tissues, LPS treatment reduced activity of HDAC1 and HDAC2 by 19.5% and 22% compared to the control group (Fig. 3B). Similar results were also observed in cultured myofibroblasts. LPS exposure decreased the protein level and activity of HDAC1/2 compared with the PBS treated group (Fig. 3C, D).

### Overexpression of HDAC2, but not HDAC1, repressed LPS-induced TGF-α expression

**Figure 4:**
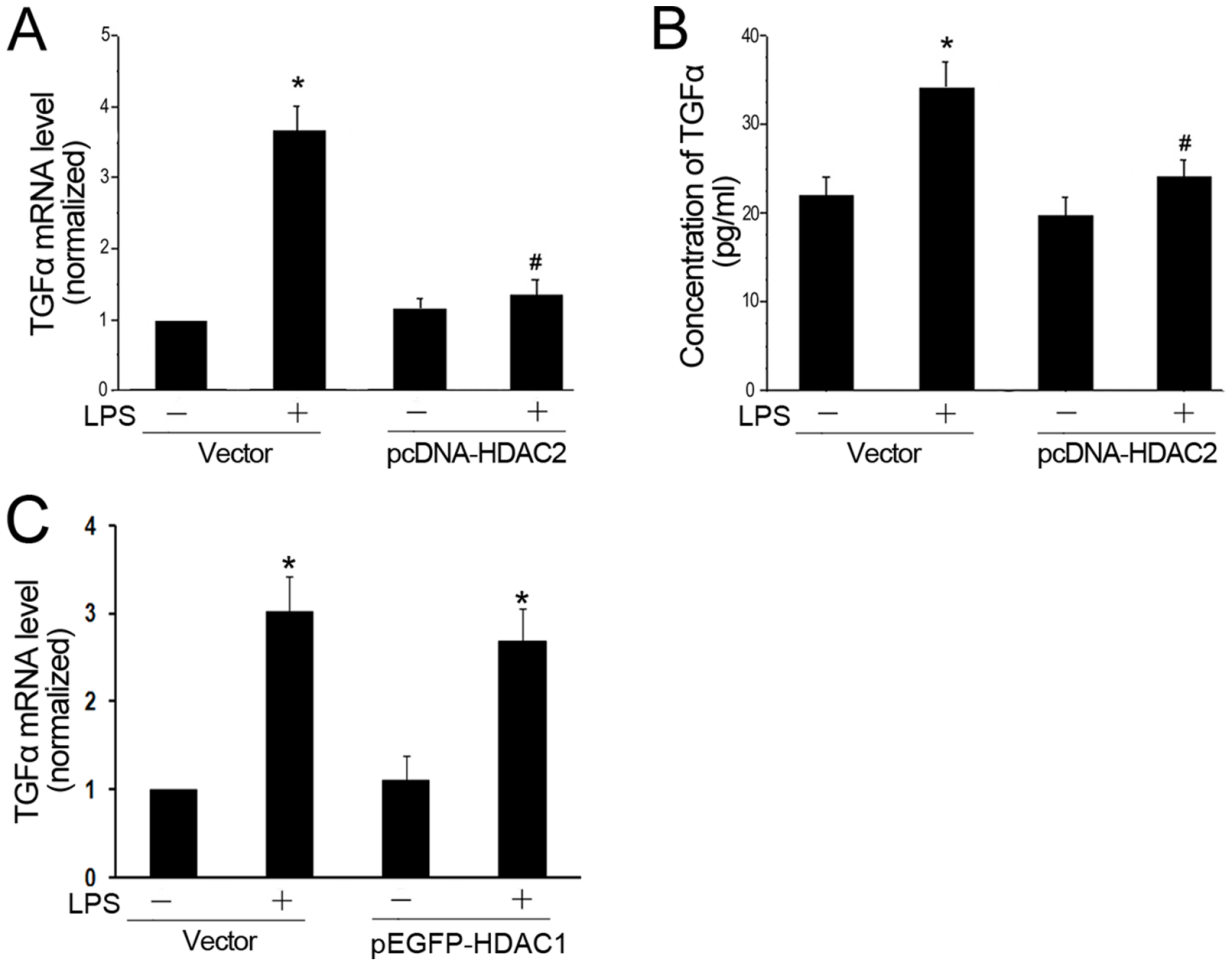
Overexpression of HDAC2 represses LPS-induced expression of TGF-α. Cells were transfected with the pcDNA-HDAC2 and a control vector for 24 h before exposure to LPS for 1 (A) or 16 (B) hours. A: TGF-α mRNA levels in myofibroblasts. B: TGF-α in supernatant of myofibroblasts. C: Cells were transfected with the GFP-tagged wild-type HDAC1 and a control vector for 24 h before exposure to LPS for 1 hour. TGF-α mRNA levels in myofibroblasts. Results from 3 separate experiments were pooled and the mean ± SE was calculated. * *P*<0.05, significantly different from vector control group; # *P*<0.05, significantly different from LPS plus vector control group.

HDAC2 overexpression was used to further examine whether HDAC2 is a key regulator of TGF-α expression. Myofibroblasts were transfected with pcDNA-HDAC2 plasmid or a control vector. LPS-induced TGF-α expression was reduced in cells overexpressing HDAC2 compared to those transected with control vectors (Fig. 4A, B). In contrast, overexpression of HDAC1 had no effect on the level of TGF-α induced by LPS (Fig. 4C).

### Both HDAC inhibitor TSA and HDAC2 knockdown increased TGF-α expression

**Figure 5:**
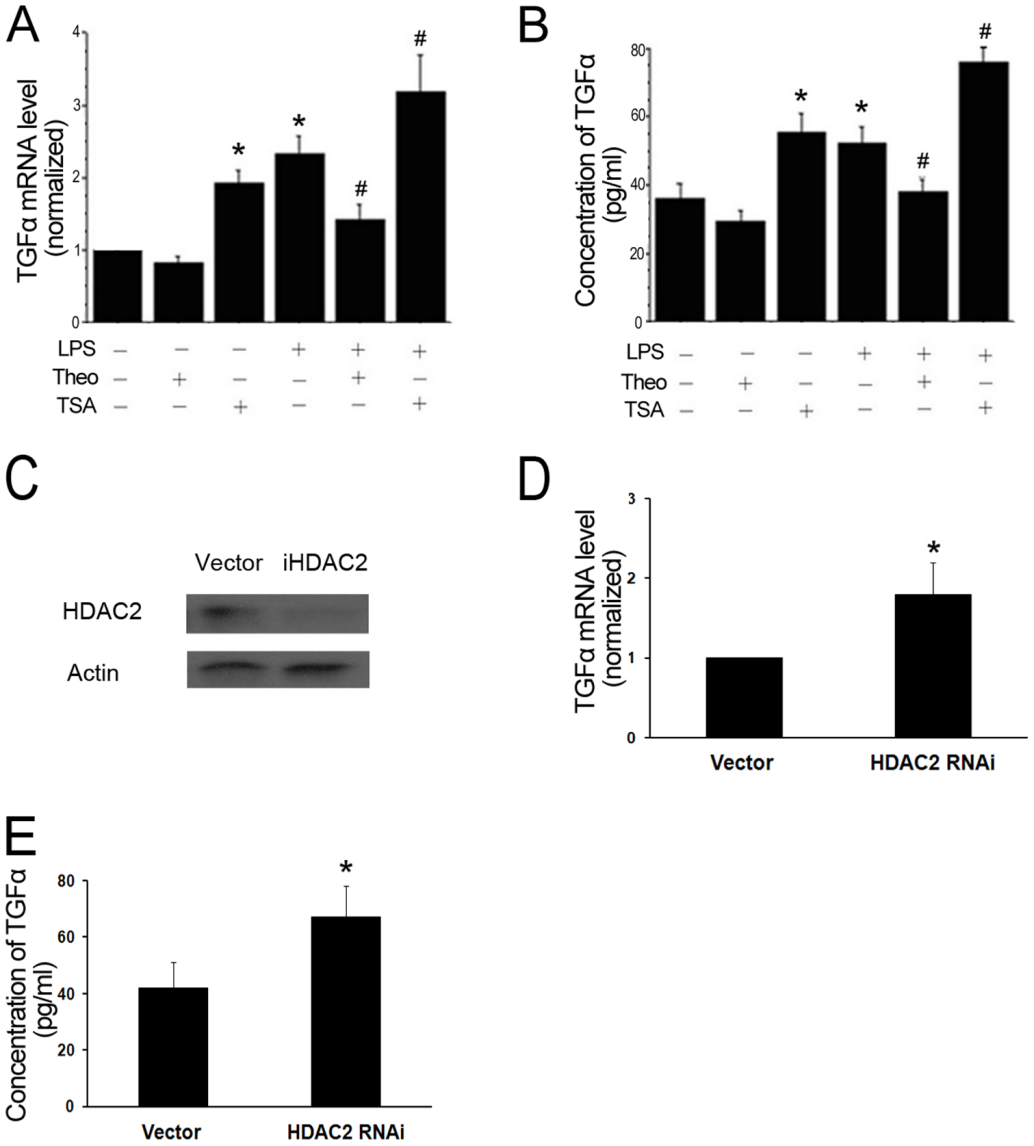
HDAC2 inhibition increased expression of TGF-α. A–B: TSA treatment caused increased expression of TGF-α. Myofibroblasts were treated with theo (theophylline, 10 μM) or TSA (Trichostatin A, 10 ng/ml) for 30 min before exposure to LPS for 1 (A) or 16 (B) hours. C: The RNAi plasmids of iHDAC2 or pPGK-super empty vector were transfected into cells and identified by immunoblotting. D–E: Suppressing HDAC2 with siRNA resulted in increased TGF-α expression. Cells were transfected with either control-vector or HDAC2 siRNA. Cells were cultivated up to 48 h. TGF-α mRNA levels were measured by real time PCR; TGF-α level in supernatant of myofibroblasts was assayed by ELISA. Results from 3 separate experiments were pooled and the mean ± SE was calculated. * *P*<0.05, significantly different from PBS group; # *P*<0.05, significantly different from LPS-treated group.

In order to investigate the functional role of HDAC in regulating TGF-α expression, we treated myofibroblasts with TSA, a potent inhibitor of the HDAC family [24]. Treatment of cultured cells with TSA increased TGF-α mRNA level (Fig. 5A). Consistently, the TGF-α protein measured by ELISA showed a similar increase (Fig. 5B). To further confirm the above results, HDAC2 RNAi plasmid (HDAC2i) was transfected into cells (Fig. 5C). Silencing of HDAC-2 by siRNA significantly increased TGF-α expression (Fig. 5D, E). These results provide evidence for the involvement of HDAC2 in regulating TGF-α expression.

### Theophylline restored HDAC2 activity, decreased TGF-α expression, and improved alveolar development in LPS-exposed rats

**Figure 6:**
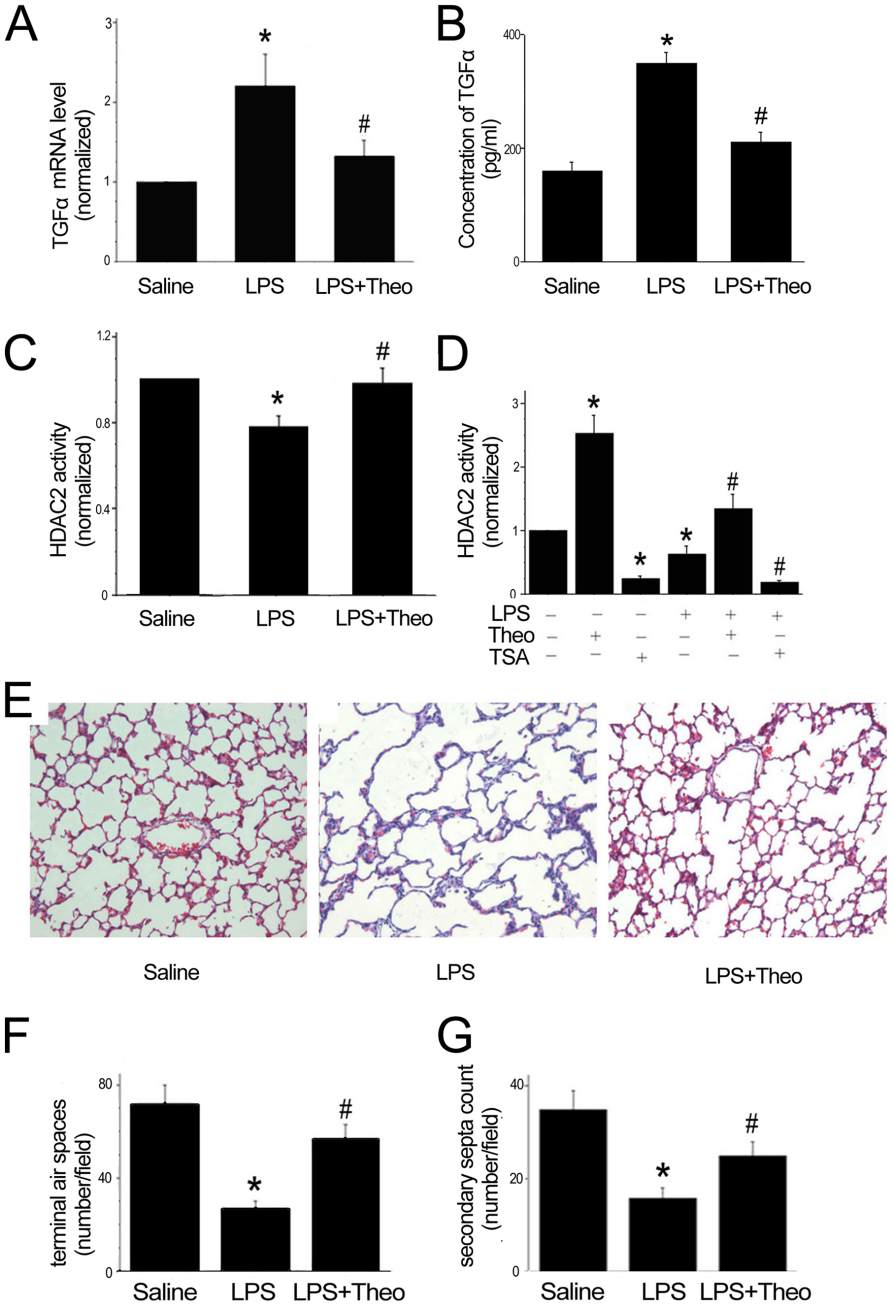
Theophylline restored lung development impaired by LPS. TGF-α mRNA (A) and protein (B) levels in the lung of neonatal rats treated with theophylline, LPS or saline. C: The activities of HDAC2 were significantly increased in rat lungs treated with theophylline for 7 days. D: The activity of HDAC2 in myofibroblasts was increased by theophylline treatment and decreased by TSA or LPS. E: Lung tissues were stained with H&E. Pregnant rats were injected with LPS and neonatal rats were injected with theophylline. F: Terminal air spaces were significantly fewer in LPS-treated rats, a result improved by theophylline treatment. G: The secondary septa count was significantly less in the LPS exposed group and improved in the theophylline group. Results from 3 individual experiments were pooled and the mean ± SE was shown. * *P*<0.05, significantly different from saline or PBS group; # *P*<0.05, significantly different from LPS-treated group.

Having shown HDAC2 regulation of TGF-α expression both *in vivo* and *in vitro*, we further tested whether restoring HDAC activity could rescue lung development. We found that a theophylline injection restored HDAC2 activity and decreased the expression level of TGF-α in both lung tissues and myofibroblasts treated with LPS (Fig. 6A–D). LPS injection led to enlargement of alveoli and a decrease in secondary septa at P7, while treatment with theophylline effectively ameliorated the impairment caused by LPS (Fig. 6E). Co-treatment with theophylline and LPS increased terminal air space compared to LPS treated alone at P7 (Fig. 6F). The number of secondary septa in LPS-treated pups was also increased after theophylline treatment (Fig. 6G). Our results indicate that alveolar development arrested by LPS exposure is partially rescued by the HDAC agonist theophylline.

## Discussion

BPD is characterized by arrested alveolarization, fewer and larger alveoli with decreased alveolar surface area [25]. Based on our research, intra-amniotic injection of LPS leads to enlarged alveoli with decreased terminal air space and secondary septa, a phenotype similar to BPD. Mechanical ventilation, hyperoxia exposure and infection are all common causes of BPD. Additionally, intra-uterine inflammation, maternal chorioamnionitis and deregulation of pro- and anti- inflammatory cytokines play important roles in disease progression [26]–[29]. Tremendous evidence supports antenatal activation of pro-inflammatory pathways after exposure to chorioamnionitis initiates an inflammatory process. Consequently the fetus is exposed to the maternal responses, such as overexpression of cytokines, lipid mediators or chemokines, present in the maternal circulation which potentiates postnatal secondary strikes on neonatal lungs, such as hyperoxia and mechanical ventilation [5], [12]. Prince [30] observed that LPS prevented saccular airway branching in both fetal mice and cultured fetal mouse lung explants. These data suggest that inflammatory signaling interacts with developmental pathways, interfering with processes required for branching morphogenesis. However, the mechanisms that lead to pathophysiological changes have not yet been understood.

Many researchers have suggested that multiple cytokines must participate in the development of BPD [31]. Inflammatory cytokines such as interleukin-8 (IL-8), macrophage inflammatory protein-2 (MIP-2) and tumor necrosis factor-alpha (TNF-a) have been shown elevated in tracheal aspirates of neonates which later develop BPD [32]. Previous studies also demonstrated TGF-α was induced by pro-inflammatory cytokines and hyperoxia [33]–[35]. In our study, we used LPS-treated myofibroblasts and found that TGF-α expression was increased. The same results were found in the lung of newborn rats exposed to LPS. These results suggest that myofibroblasts must also participate in inflammation induced lung injury.

Because TGF-α is regulated primarily at the level of gene transcription, elucidating the factors involved in increasing transcription of the TGF-α gene may be critical to understanding LPS-induced arrest of alveolarization. Based on mounting evidence that environmental triggers exert changes in lung architecture through altered epigenetic mechanisms [19], [36], we hypothesized that LPS disrupts alveolar development through an imbalance of histone deacetylases (HDACs). HDAC1/HDAC2 are crucial for embryonic development and regulates fundamental biological processes such as cellular proliferation, differentiation, and survival via genomic and non-genomic effects [37]. HDACs have the ability to deacetylate non-histone proteins such as nuclear factor κB (NF-κB) which play a critical role in inflammatory signaling pathways [15]. Londhe et al [38] utilized an established newborn mouse model to show that the mechanism by which hyperoxia induces alveolar hypoplasia was through decreased HDACs and increased p21, which was consistent with our previous study [39]. In this study, our results showed that LPS reduced HDAC1/HDAC2 expression and activity. We also found that preservation of HDAC activity by theophylline treatment improved alveolar development. These data suggest that HDAC1/2 play an important role in arresting alveolar development. Further study revealed that LPS-induced increase of TGF-α was dependent on HDAC2, but HDAC1-independent. Although we demonstrate that the inhibition of HDAC2 is required for LPS-induced TGF-α expression, it is currently not known whether other proinflammatory cytokines are also affected by HDAC2. It is very likely that a reduction in HDAC2 could affect the regulation of several genes in our model and contribute to the pathogenesis of BPD. In addition, it is unclear how HDAC1 influences alveolar developmental.

Using siRNA to reduce HDAC2 level, we show that HDAC2 plays a role in the transcriptional regulation of TGF-α in myofibroblasts. HDACs are epigenetic regulators that are important for the control of proteins function. Many of these proteins are transcription factors, such as p53, C/EBPβ, NF-κB and STATs [40]. Therefore the mechanisms regulating the interactions between HDAC2 and TGF-α could be due to a direct modulation of the “histone code” or the consequence of indirect modulation of signaling pathways and transcription factor activities. Further studies are needed to clarify the mechanisms how HDAC2 regulates the TGF-α/EGFR pathway in newborns suffering from maternal chorioamnionitis exposure.

In summary, these data suggest that LPS causes myofibroblasts dysfunction, an important target cell type in BPD. This change is at least partially due to the increased TGF-α level in response to LPS stimulation. The down-regulation of HDAC2 in response to LPS stimulation contributed to the up-regulation of TGF-α production. Our results may provide potential therapeutic targets in BPD.
